# Morpho‐Reproductive Stability of Rice Landraces Under Water Deficit Across Agroecological Zones of Far‐West Nepal

**DOI:** 10.1002/pei3.70170

**Published:** 2026-06-10

**Authors:** Nabin Lamichhane, Urmila Dhami, Sudan Bhandari, Gunanand Pant, Lal Bahadur Thapa, Chandra Prasad Pokhrel, Ram Kailash Prasad Yadav

**Affiliations:** ^1^ Central Department of Botany, Institute of Science and Technology Tribhuvan University Kirtipur Kathmandu Nepal; ^2^ Department of Biology Kailali Multiple Campus, Far Western University Dhangadhi Nepal

**Keywords:** drought resilience, drought stress, morphological parameters, rice landraces, yield

## Abstract

Rice landraces represent a reservoir of genetic diversity with potential for enhancing drought tolerance abilities. This study evaluated the morphological and reproductive responses of twelve rice landraces to drought stress from three agro‐ecological regions (low land Tarai, Inner‐Tarai, and Mid‐hill) Nepal. The landraces were grown under the treatments of regular irrigation, intermittent irrigation, and without irrigation by water after seedling establishment in the farmers' field. The traits such as root and shoot length, shoot‐to‐root ratio, panicle length, and yield were measured during harvesting time. The data were analyzed using two‐way analysis of variance, multivariate principal component analysis, and Pearson correlations to know the variations among the landraces and drought treatments across agro‐ecological regions. Trait responses varied significantly among landraces across all regions, whereas treatment effects were not significant in most cases. Traits such as shoot‐to‐root ratio, panicle length, and grain weight showed relative stability across treatments. However, drought reduced tiller number in the rice Anjana and Lalchand, decreased shoot length in Chamade and Ratomarso, and lowered total grains per panicle in Jhini and Shanti. Principal Component Analysis (PCA) indicated that variation was primarily associated with the landraces rather than treatment effects, with the first two principal components explaining 49.1%, 48.6%, and 43.9% of total variance in the Mid‐hill, Inner‐Tarai, and Tarai regions, respectively. Yield was positively associated with root length, grain weight, and grains per panicle. Cluster analysis indicated grouping of landraces based on agro‐ecological origin, suggesting adaptation to specific environmental conditions. This study identifies locally adapted landraces with consistent performance across different water treatments and provides candidate material for further evaluation and utilization for drought‐focused breeding programs.

## 1 Introduction

Rice ( *Oryza sativa* L.) is one of the most important cereal crops globally and serves as a staple food for the majority of the world's population with its significant role in global food security (Rezvi et al. 2023; Verma et al. 2021). Ongoing climate change has intensified the frequency and severity of drought events in South Asia, where rice is highly dependent on seasonal rainfall (Chandrasekara et al. 2021; Hussain et al. 2020; Kumar et al. 2023). Drought stress adversely affects rice growth, physiology, and reproductive development, leading to affecting the morphological, biochemical traits, and overall yield (Kim et al. 2020; Panda et al. 2021). Therefore, drought is considered one of the major constraints limiting rice productivity under rainfed and water‐limited agriculture systems (Hussain et al. 2020; Kumar et al. 2023). In such a scenario, identifying rice genotypes capable of maintaining growth and reproductive stability under water‐deficit conditions has become urgent for sustainable rice production and climate adaptation.

Rice landraces are known for wide diversity, featuring numerous distinct varieties and races within both irrigated and upland agroecosystems (Hour et al. 2020; Zhang et al. 2013). They are valuable genetic resources because they have evolved under long‐term cultivation across diverse environmental conditions and management practices resulting in high genetic and phenotypic variability (Marone et al. 2021; Pusadee et al. 2014). Through these selections, rice varieties have been evolved to exhibit traits that are better suited to environmental stresses such as drought, floods, pests, and diseases (Vaughan et al. 2008; Vikram et al. 2016; Baniya et al. 2022).

Nepal is characterized with agroecological gradients ranging from lowland Tarai to mid‐hills. Across these heterogeneous landscapes, farmers have traditionally maintained a wide diversity of rice landraces adapted to local environments (Baniya et al. 2022). Also, rice cultivation in Nepal is strongly linked to the culture, national food security, demographic dynamics, and climate vulnerability (Lamichhane et al. 2024). Studies have highlighted that about 2500 rice landraces and 8389 rice accessions are reported from Nepal (Joshi 2017). Despite this diversity, efforts to characterize and identify valuable adaptable traits in diverse environmental conditions are limited. Evaluating the landraces from diverse environmental conditions may therefore provide important insights into morpho‐reproductive traits associated with adaptation to water‐limited environment.

Previous studies on rice drought adaptation have focused on physiological, biochemical and molecular responses under controlled or experimental conditions, including osmotic adjustment, antioxidative defense mechanism and root architectural traits associated with water stress tolerance (Kim et al. 2020; Melandri et al. 2021; Panda et al. 2021). Several studies have also evaluated the drought tolerance through various indices (Kandel et al. 2022; Lamichhane et al. 2024) and some has also evaluated drought related traits and allele diversity in traditional rice germplasm and breeding lines (Mishra et al. 2018). However, despite the rich diversity of rice landraces in Nepal, comparative field‐based assessment of morpho‐reproductive response under water stress across agroecological regions remain scarce. Such evaluations are important because plant responses to drought are strongly influenced by local environmental conditions and genotype‐environment interactions (Gratani 2014; Khadka et al. 2020). Thus, assessment of landraces under water stress has been necessary to better understand their potential adaptive response.

This study evaluated the morpho‐reproductive response of selected 12 rice landraces under water stress across three agroecological regions of far‐west Nepal. This study assessed variation in growth traits, biomass allocation and reproductive performance under regular irrigation, intermittent irrigation and no irrigation conditions. As we selected the landraces perceived by farmers as drought adapted with the hypothesis that they would maintain greater morpho‐reproductive stability under water‐deficit conditions. By integrating field‐based evaluation across Tarai, Inner‐Tarai and Mid‐hills agroecological regions, this study provides evidence that the farmer‐selected rice landraces maintain relative morpho‐reproductive stability under water‐deficit conditions, supporting their further evaluation as potential genetic resources for climate‐resilient rice improvement.

## 2 Materials and Methods

### 2.1 Experimental Sites and Rice Landraces

**FIGURE 1:**
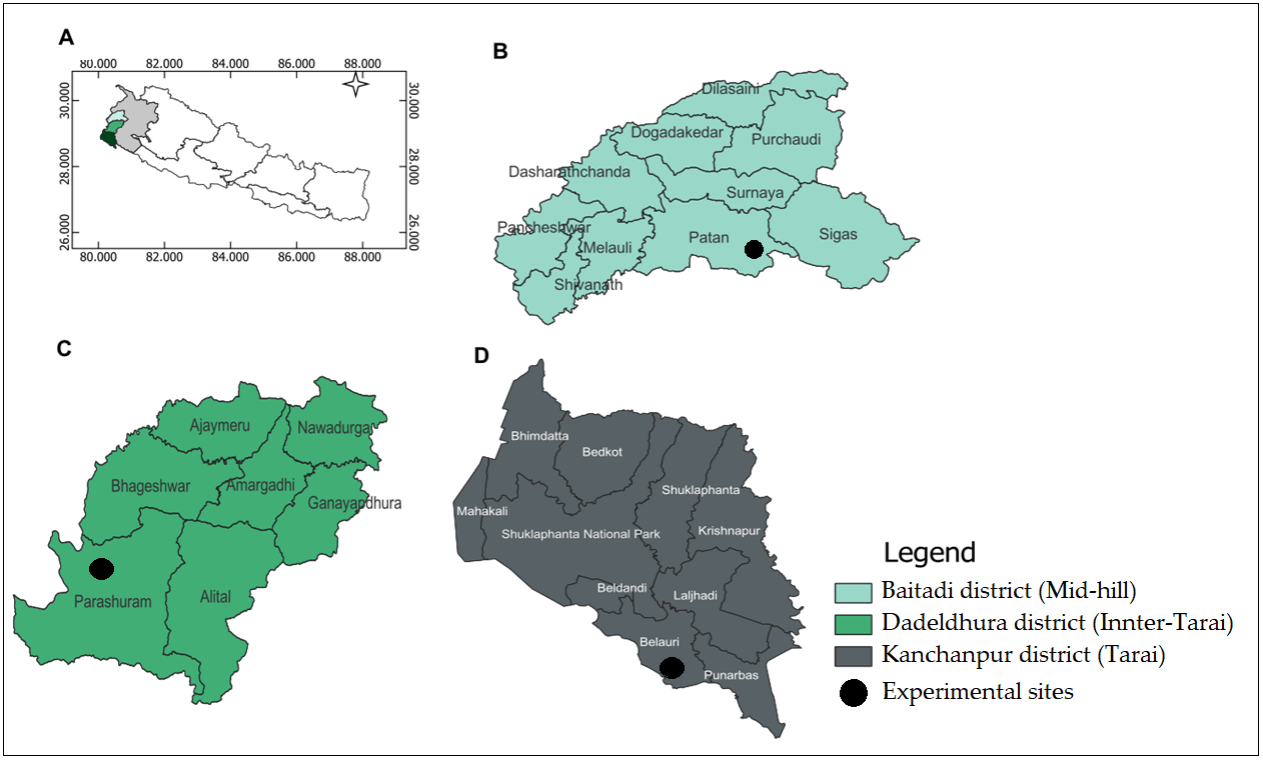
Study area maps and experimental station locations. (A) Nepal showing the far‐west region. (B) Mid‐hill, Baitadi, (C) Inner‐Tarai, Dadeldhura and (D) Tarai, Kanchanpur. The points indicate experimental sites.

Field experiments were conducted in three agro‐ecological regions of the Far‐western region of Nepal (Figure 1). The sites were located in Belauri Municipality (Tarai region), Parshuram Municipality (Inner‐Tarai region) and Patan Municipality (Mid‐hill region) (Table 1). Each agro‐ecological region is diverse due to microclimates and elevation. Tarai is low land with fertile plain area in southern side. The Inner‐Tarai is located between within Chure hills above Tarai region, which has humid subtropical climate. The Mid‐hill at higher elevation towards the northern side, which is relatively colder than the Tarai and Inner‐Tarai. The research station Tarai (Kanchanpur) exhibits average annual maximum and minimum temperatures 27.1°C with average annual rainfall 1593. Average annual maximum temperature of Inner‐Tarai (Dadeldhura) is 20.8°C with annual average rainfall of 1523 mm. Similarly, Mid‐hill (Baitadi) experiences a mean temperature of 16.9°C with an average rainfall of 1156 mm.

**TABLE 1:** Details of experimental plots across agro‐ecological regions.

S. N.	Agro‐ecological regions	District and municipality	Geographical coordinates	Altitude (masl)	Rice landraces
1	Mid‐hill	Baitadi, Patan	29.478°N–80.617°E	1666 m	Ratomarso, Temase, Jhumke, Chamade
2	Inner‐Tarai	Dadeldhura, Parshuram	29.160°N–80.286° E	353 m	Shanti, Jhini, Badebudo, Chiudi
3	Tarai	Kanchanpur, Belauri	28.696°N–80.376°E.	180 m	Sauthayari, Ghiupuri, Lalchand, Anjana

Twelve local rice landraces were selected for this study from three agro‐ecological zones (four landraces from each region) as shown in Table 1. The landraces were selected based on recommendations from the local farmers. The landraces Chamade, Jhumke, Ratomarso, and Temase were selected from the Mid‐hills; Chiudi, Jhini, Batebudo, and Shanti from the Inner‐Tarai; and Anjana, Lalchand, Ghiupuri, and Sauthyrai from the Tarai region. According to the local farmers, these landraces were among the best preferred for water‐stressed conditions.

### 2.2 Seedling Preparation, Transplantation and Treatments

Seedlings of each rice landrace were grown in nursery beds at each experimental site. For the transplantation of 30‐day‐old seedlings, plots were prepared (each 2 × 1.5 m size) following traditional practice as adopted by local farmers. After seedling transplantation, all the plots were irrigated for 15 days. After that, three water stress levels were maintained for each rice landrace in each experimental site as follows:

- Regular irrigation: Five plots for each rice variety were continuously irrigated. This treatment assigned as the control plots. The moisture level in these plots was maintained at 100%.
- Intermittent drought: Five plots were irrigated for a week and re‐irrigated intermittently when approximately 50% of plant wilting was observed after stopping irrigation. It was assigned as intermittent drought. The soil moisture level in these plots ranged from 25% to 35% during wilting.
- Complete drought: Five plots were irrigated for a week and then irrigation was stopped. The plots experienced soil moisture level < 15%. This treatment was assigned as complete drought.

Soil moisture was measured using digital sensors (model: WTPH01803, probe length 20.5 cm, 35 × 8 × 6 cm) at randomly selected location within each plot. The sensor was inserted to a depth of 10–15 cm and readings were recorded twice per week. Rainout shelters were prepared to protect the plots from rainfall. Altogether, there were 180 plots (12 varieties × 3 treatments × 5 replications). Each treatment consisted of five replicated plots (2 × 1.5 m) per landrace. The plots were distributed in a randomized complete block design with split experimental sites (separate plots for each water regime). Each agro‐ecological zone was analyzed independently because different landraces were evaluated in each region.

### 2.3 Plant Sampling and Measurement of Growth Parameters

Rice plants grown in plots were sampled for measuring growth parameters during the harvesting period. Morphological parameters (number of tillers, shoot and root length, and biomass) were measured. Shoot length was measured from the base of the shoot up to the longest leaf or panicle. Individual plant samples were uprooted carefully, washed of the soil from roots, and root length was measured. The fresh weights of both shoot and roots were taken and oven‐dried at 80°C until the constant weight for the estimation of dry weight. The reproductive parameters included panicle length, total grains per panicle, and yield (kg/plot).

### 2.4 Statistical Analysis

Data for two successive years 2022 and 2023 were used for statistical analysis. Two‐way analysis of variance (ANOVA) was used to evaluate the effect of the variety and treatment in each agro‐ecological zone. Differences between treatments within each variety were assessed using pairwise post hoc multiple comparisons of estimated marginal means with Tukey adjustment at *p* < 0.005. Correlation analysis was done to evaluate the relationship between yield and other morphological parameters. For all the analyses, *p* < 0.05 was considered statistically significant.

Principal component analysis (PCA) was used to assess the extent of demarcation between the varieties and treatment and the contribution of each parameter for that demarcation, using the *FactomineR* package. Hierarchical cluster analysis was done to know the clustering of the landraces based on all measured parameters after doing PCA. All the statistical analyses were carried out by using R Studio (R Core Team 2024).

## 3 Results

### 3.1 Tiller Number, Shoot and Root Length

**FIGURE 2:**
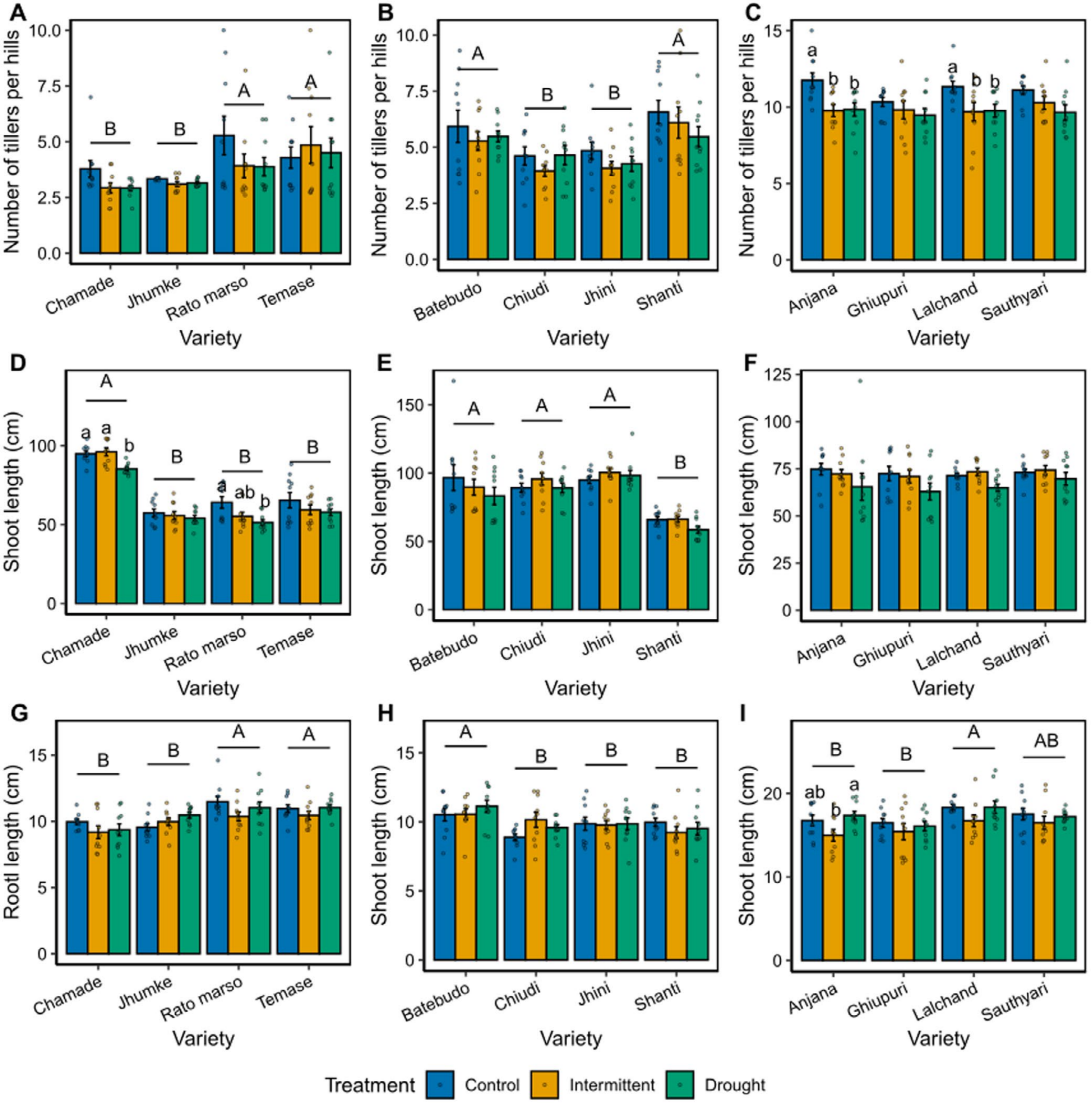
Number of tillers (A–C), shoot length (D–F), and root length (G–I) across different levels of water treatments and landraces. The bar graphs display the mean value ± SE. Different uppercase letters indicates significant difference between the varieties and lowercase different letters indicated difference between treatments in same variety based on post hoc multiple comparisons of estimated marginal means with Tukey adjustment at *p* < 0.005. Jittered points represent individual observations.

In the Mid‐hill and Inner‐Tarai, the number of tillers varied significantly with the landraces (Figure 2A,B), but there was no significant variation due to treatment or the interaction between landrace and treatment (*p* > 0.05) (Table 2). In the Tarai, the number of tillers decreased significantly due to drought in the Anjana and Lalchand landrace (*p* < 0.01), but there was no significant difference among other landraces (*p* > 0.05) (Table 2, Figure 2C).

**TABLE 2:** Results of two‐way analysis of variance (ANOVA) for morphological parameters across different agro‐ecological regions, showing effect of treatments (T) and landrace (L).

Parameters	Mid‐hill	Inner‐Tarai	Tarai
Number of tillers	T: F = 1.55^ns^	T: F = 2.29^ns^	T: F = 12.25***
	L: F = 6.84***	L: F = 10.23***	L: F = 0.96^ns^
	T × L: F = 0.88^ns^	T × L: F = 0.44^ns^	T × L: F = 0.63^ns^
Shoot length	T: F = 9.55***	T: F = 1.71^ns^	T: F = 5.61**
	L: F = 123.91***	L: F = 33.06***	L: F = 0.58^ns^
	T × L: F = 1.25^ns^	T × L: F = 0.81^ns^	T × L: F = 0.22^ns^
Root length	T: F = 2.88^ns^	T: F = 0.27^ns^	T: F = 5.81**
	L: F = 12.70***	L: F = 5.79**	L: F = 4.52**
	T × L: F = 1.4^ns^	T × L: F = 1.27^ns^	T × L: F = 0.40^ns^

*Note:* Significant effects are marked as *p < 0.05, **p < 0.01, ***p < 0.001, and “ns” denotes non‐significant (*p* > 0.05) results. T × L represents interactive effect of treatment and landraces.

Tarai landraces did not exhibit variation in shoot length among them, while the length differed among the landraces within Inner‐Tarai and Mid‐hill (*p* < 0.001) (Table 2). The landraces Chamade has longer shoots in Mid‐hills (Figure 2D), while in Inner‐Tarai Shanti has shorter shoot length (Figure 2E). Drought significantly decreased the shoot length of Chamade and Ratomarso landraces in the Mid‐hills (*p* < 0.01) (Figure 2D). The length in the other varieties remained consistent across the treatments (*p* > 0.005) (Figure 2D–F).

Landrace‐wise variation was exhibited in the root length in all agro‐ecological regions (*p* < 0.01); however, the interaction between treatment and landrace showed no effect (Table 2). Anjana variety from the Tarai region has a significantly longer root length in complete drought than in the intermittent drought.

### 3.2 Moisture Content in Roots and Shoots and Shoot‐To‐Root Dry Weight Ratio

**FIGURE 3:**
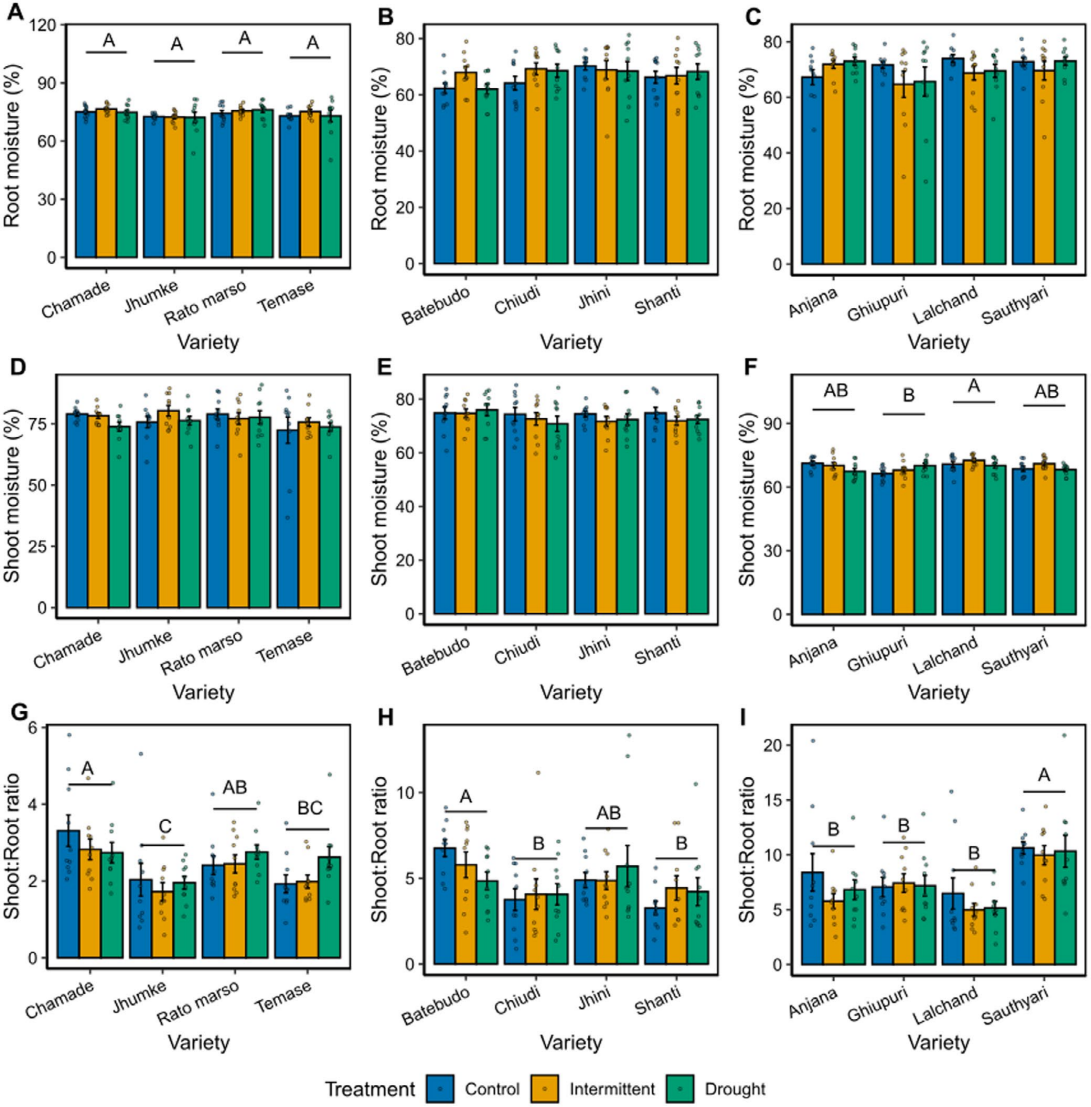
Root moisture (A–C), shoot moisture (D–F), and shoot‐to‐root ratio (G, H, I) across different levels of water treatments and landraces. The bar graphs display the mean value ± SE (standard error). Different uppercase letters indicate significant difference between the varieties and lowercase different letters indicated difference between treatments in same variety based on post hoc multiple comparisons of estimated marginal means with Tukey adjustment at *p* < 0.005. Jittered points represent individual observations.

Water stress didn't significantly affect the root and shoot moisture content in tested rice landraces (Table 3). Moisture content also did not differ significantly among landraces (*p* > 0.05), except in the Mid‐hill and Tarai regions where significant differences were observed (Figure 3). However, post hoc comparison showed no difference in mid‐hills landraces (Figure 3A). In Tarai, higher shoot moisture was observed in Lalchand (Figure 3F). The interaction of treatment and landrace was not found to have a significant effect on the moisture content except for the Tarai landraces (Table 3). The shoot‐to‐root dry weight ratio varied among landraces but was not significantly influenced by water stress across all regions (Figure 3G–I).

**TABLE 3:** Results of two‐way analysis of variance (ANOVA) for plant water status and biomass allocation traits across agro‐ecological regions, showing the effect of treatments (T) and landrace (L).

Parameters	Mid‐hill	Inner‐Tarai	Tarai
Root moisture %	T: F = 0.70^ns^	T: F = 1.03^ns^	T: F = 0.94^ns^
	L: F = 2.77*	L: F = 2.22^ns^	L: F = 1.48^ns^
	T × L: F = 0.27^ns^	T × L: F = 0.80^ns^	T × L: F = 1.20^ns^
Shoot moisture (%)	T: F = 1.06^ns^	T: F = 1.02^ns^	T: F = 1.79^ns^
	L: F = 1.66^ns^	L: F = 0.99^ns^	L: F = 3.46*
	T × L: F = 0.65^ns^	T × L: F = 0.29^ns^	T × L: F = 2.19*
Shoot root ratio	T: F = 1.03^ns^	T: F = 0.03^ns^	T: F = 1.23^ns^
	L: F = 8.47***	L: F = 5.03**	L: F = 11.51***
	T × L: F = 1.03^ns^	T × L: F = 1.05^ns^	T × L: F = 0.90^ns^

*Note:* Significant effects are marked as *p < 0.05, **p < 0.01, ***p < 0.001, and “ns” denotes non‐significant (*p* > 0.05) results. T × L represents the interactive effect of treatment and landraces.

### 3.3 Reproductive Parameters

**FIGURE 4:**
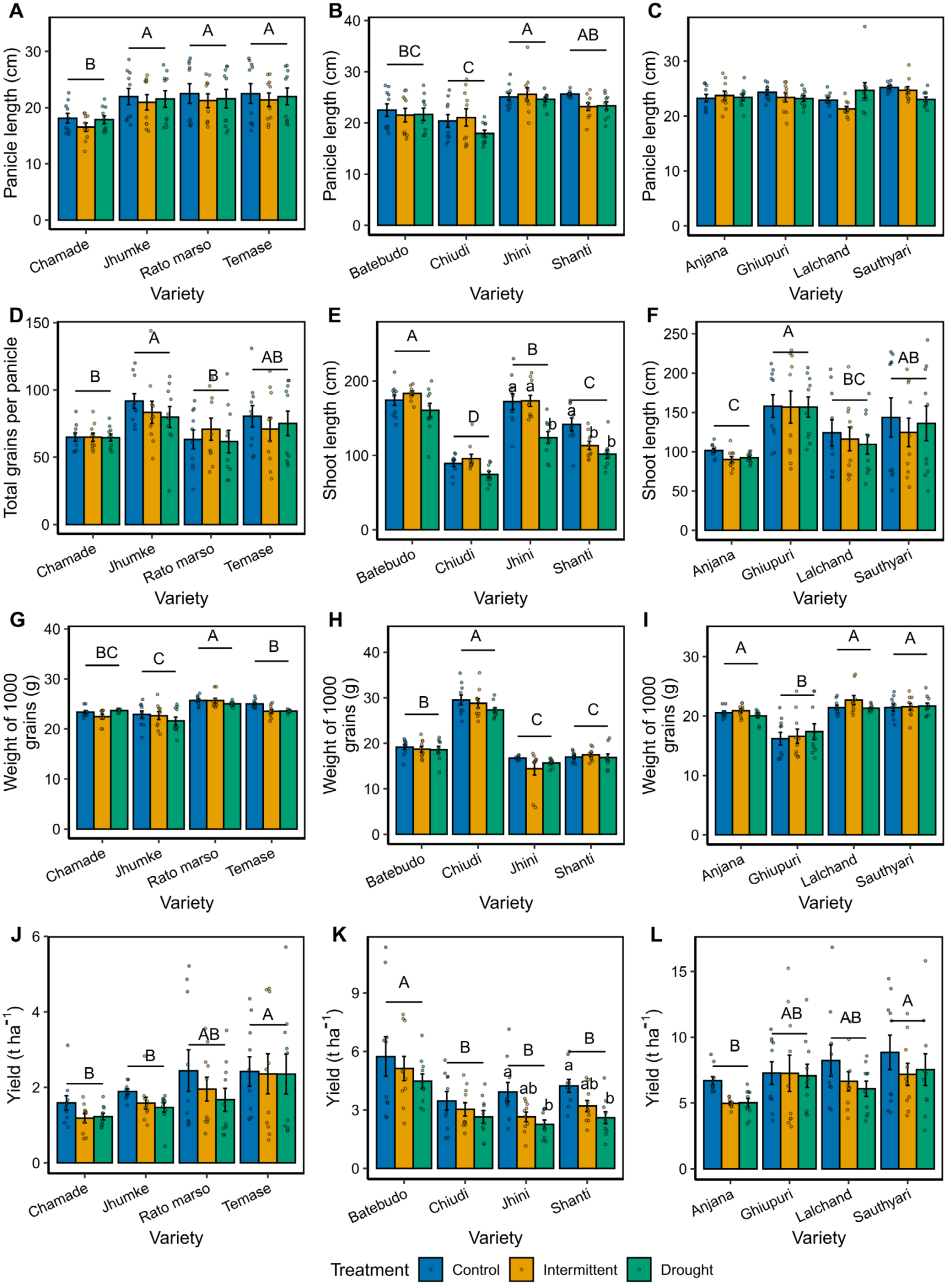
Panicle length (A–C), grain number per panicle (D–F), grain weight (G–I), and yield (t/ha) (J–L) across different levels of water treatments and landraces. The bar graphs display the mean value ± SE (standard error). Different uppercase letters indicate significant difference between the varieties and lowercase different letters indicated difference between treatments in same variety based on post hoc multiple comparisons of estimated marginal means with Tukey adjustment at *p* < 0.005. Jittered points represent individual observations.

Two‐way ANOVA shows that panicle length was different among the varieties in mid‐hills and Inner‐Tarai, whereas in Tarai they were not significantly different (Table 4). Chamade landrace of the Mid‐hill showed shorter panicle length compared to other landraces (Figure 4A). Similarly, Jhini had higher panicle length and Chiudi had shorter in Inner‐Tarai. The landraces of Tarai did not show variation in the length with water stress and varieties, but the effect of interaction between treatment and interaction was found to be significant (*p* < 0.01).

**TABLE 4:** Results of two‐way analysis of variance (ANOVA) analysis of reproductive parameters in different agro‐ecological zones, showing effect of treatments (T) and varieties (V).

Parameters	Mid‐hill	Inner‐Tarai	Tarai
Panicle length	T: F = 0.85^ns^	T: F = 2.88^ns^	T: F = 0.89^ns^
	L: F = 7.37***	L: F = 12.70***	L: F = 1.98^ns^
	T × L: F = 0.03^ns^	T × L: F = 1.4^ns^	T × L: F = 2.98**
Total grains per panicle	T: F = 0.45^ns^	T: F = 21.22***	T: F = 0.46^ns^
	L: F = 5.57**	L: F = 92.54***	L: F = 8.77***
	T × L: F = 0.40^ns^	T × L: F = 2.97**	T × L: F = 0.09^ns^
Weight of 1000 grains weight	T: F = 3.12*	T: F = 2.0^ns^	T: F = 0.54^ns^
	L: F = 23.21***	L: F = 186.82***	L: F = 1.57***
	T × L: F = 1.56^ns^	T × L: F = 1.10^ns^	T × L: F = 0.54^ns^
Yield (t ha^−1^)	T: F = 0.19^ns^	T: F = 8.43***	T: F = 2.71^ns^
	L: F = 5.61**	L: F = 14.15***	L: F = 3.4*
	T × L: F = 0.21^ns^	T × L: F = 0.23^ns^	T × L: F = 0.93^ns^

*Note:* Significant effects are marked as *p < 0.05, **p < 0.01, ***p < 0.001, and “ns” denotes non‐significant (*p* > 0.05) results. T × L represents interactive effect of treatment and landraces.

In case of grains per panicle, they differ across the varieties in all agro‐ecological zones. Treatment do not have any significant effect on it in other agro‐ecological zones, while in the Inner‐Tarai they decrease due to drought (Table 4). Jhumke (Mid‐hills), Batebudo (Inner‐Tarai), and Ghiupuri (Tarai) were the varieties with highest grains per panicle in each agro‐ecological zone (Figure 4D–F).

The variation in the weight of grains was found to be strongly significant (*p* < 0.001) (Table 4) across the landraces. Water stress and its interaction with the landraces did not show significant variation in the grain weight except for water‐stressed plants of Mid‐hills (*p* < 0.05). Jhumke (Mid‐hills), Jhini, and Shanti (Inner‐Tarai) and Anjana (Tarai) were the landraces having low grain weight (Figure 4G–I). The yield (t/ha) also varied among the landraces in all agro‐ecological zones (Table 4, Figure 4J–L). In Inner‐Tarai Jhini and Shanti reduced yield under drought stress (Figure 4K).

### 3.4 Principal Component Analysis (PCA) and Cluster Analysis

**FIGURE 5:**
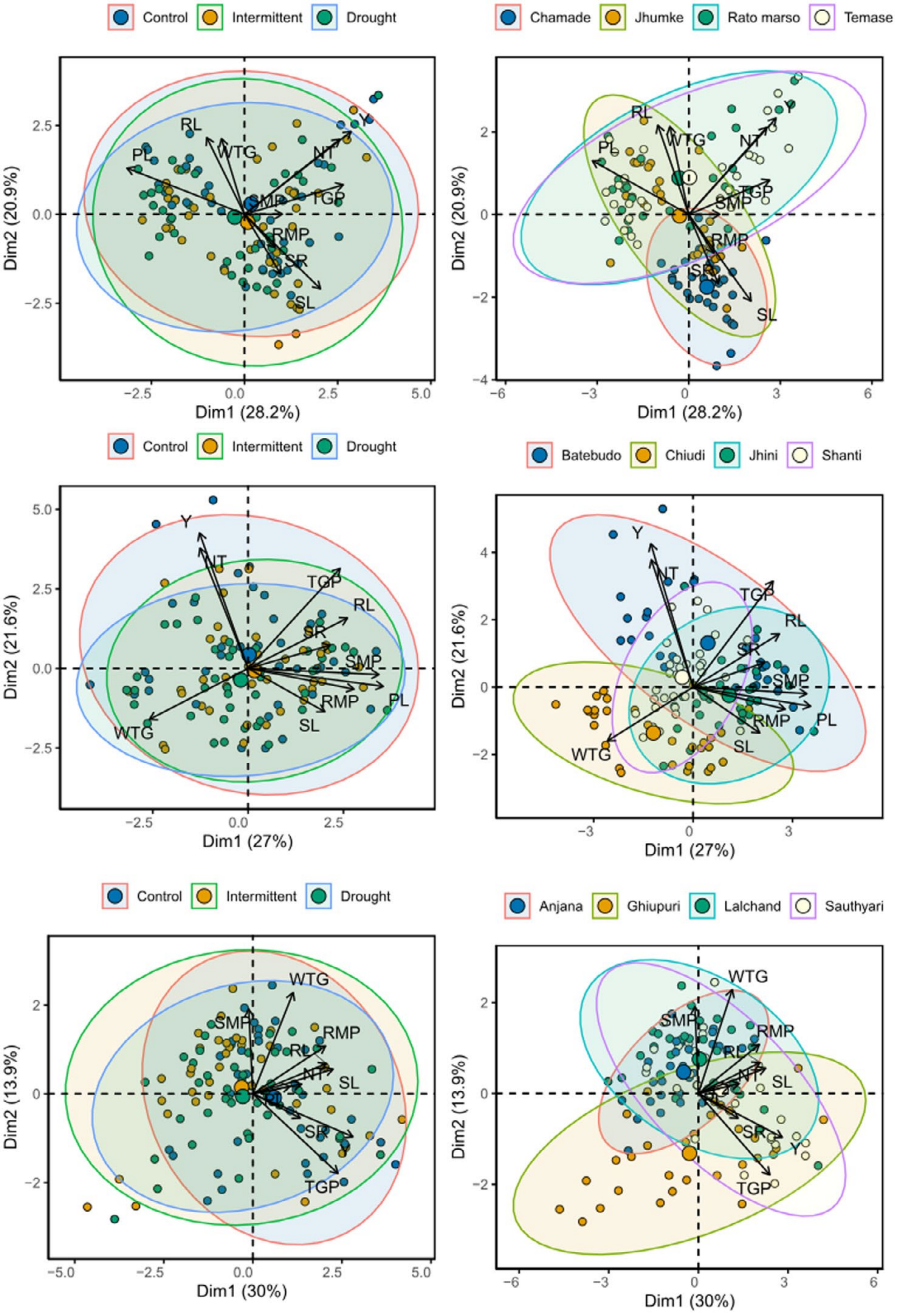
Principal component analysis (PCA) biplot illustrating the impact of landraces and treatments on the first two principal components. The first column illustrates the overall effect of treatment and the second column illustrates the varietal effect. The biplot highlights variance explained by morphological parameters and their relationships with varieties and treatments. Y = grain yield (gm), WTG = weight of thousand grains weight, NT = number of tillers, RMP = root moisture percentage, SMP = shoot moisture percentage, SL = shoot length (cm), RL = root length (cm), PL = panicle length (cm).

The results of PCA showed that varietal differences explained most of trait variation, while treatment effects were comparatively minor (Figure 5). The first two principal components explained 49.1% of the variance in the Mid‐hills, 48.6% in the Inner Tarai, and 43.9% in the Tarai. The Chamade landrace exhibited less variation in morphological characters compared to other landraces. The Ratomarso and Temase landraces exhibited overlapping characteristics with no significant variation between them. In the Inner‐Tarai, drought had no effect on the overall parameters. The Chiudi landrace was distinct from other varieties while The Batebudo landrace was more associated with high yield. In the Mid‐hill region, the Ghiupuri landrace showed wide variation, whereas the Sauthyari landrace showed less variation and was closely associated with high yield (Figure 5).

**FIGURE 6:**
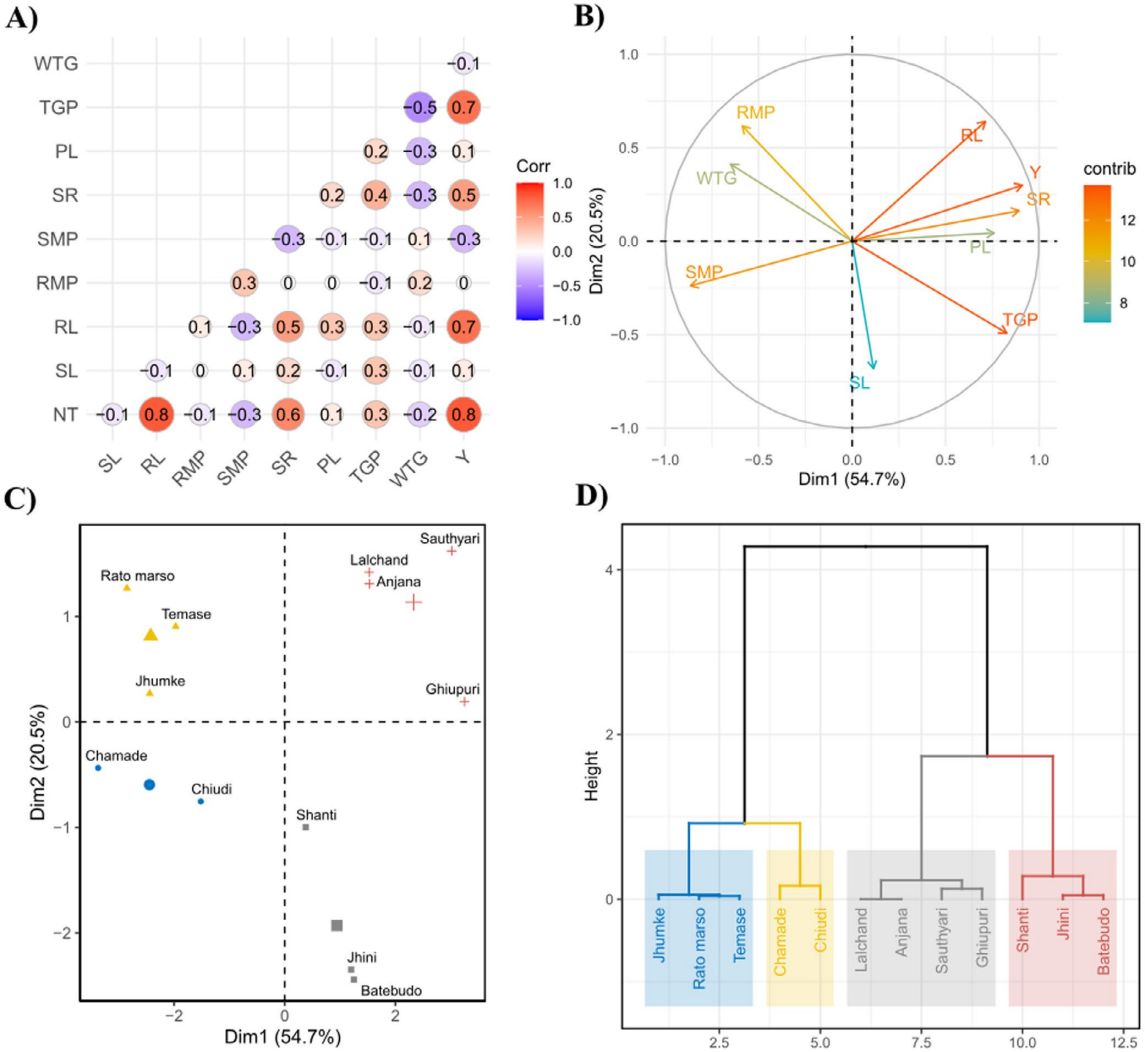
Correlation between the measured parameters (A), PCA showing the parameters' effects (B), PCA showing the distribution of varieties (C) and the hierarchical cluster dendrogram based on the PCA (D). Y = grain yield (gm), WTG = weight of thousand grains weight, NT = number of tillers, RMP = root moisture percentage, SMP = shoot moisture percentage, SL = shoot length (cm), RL = root length (cm), PL = panicle length (cm).

Combined PCA result also showed that the variation in the morphological characters was more pronouncedly explained by yield along with the root length and total grains per panicle (Figure 6A,B). The result also clearly differentiated the varieties in the PCA space (Figure 6C). The cluster dendrogram based on this PCA result of all the measured morphological parameters identified four groups. The grouping of the varieties is generally based on the agro‐ecological regions except cluster 2, where the landrace Chamade from Mid‐hill and the landrace Chiudi from the Inner‐Tarai were grouped (Figure 6D). The correlation analysis showed that the yield of landraces was positively correlated with grain weight, shoot‐to‐root ratio, root length and the number of tillers (Figure 6A).

## 4 Discussion

Landraces are the crop genotypes maintained by continuous cultivation in specific climatic, soil, and management conditions (Fischbeck 2003). This study showed that in all agroecological zones, the variation among the landraces was stronger than the effect of water treatment across most measured morpho‐reproductive traits. Although drought reduced tiller number in the Anjana and Lalchand landraces, decreased shoot length in the Chamade and Ratomarso, and lowered grains per panicle in Jhini and Shanti, other traits including shoot‐to‐root ratio, panicle length, and grain weight remained relatively stable across treatments.

Similarly, PCA indicated stronger differentiation among landraces among water treatment. This result suggests that morpho‐reproductive responses were more strongly associated with landrace identity and agro‐ecological origin. Previous studies have also reported substantial genotype‐dependent variation in rice responses to drought stress and highlighted the importance of local adaptation and phenotypic plasticity under water‐limited conditions (Mishra et al. 2018; Panda et al. 2021; Gratani 2014). The relatively limited treatment effect observed in the present study may indicate partial morpho‐reproductive stability of selected landraces under water stress.

### 4.1 Vegetative Trait Response and Biomass Allocation Under Water Stress

Vegetative traits varied among landraces rather than across water stress treatments. This indicates strong landraces specific differences in morphological performance under field conditions in all three agro‐ecological zones. Tillering is crucial for increasing yield by producing more stems, which enhances resource utilization and competition for sunlight and nutrients (Yuan et al. 2024). Tiller number remained relatively stable across treatments in most landraces, although drought significantly reduced tiller production in the Anjana and Lalchand landraces of the Tarai region. Since tiller production is closely associated with biomass accumulation and yield formation in rice (Sabetfar et al. 2013; Yan et al. 2023), the maintenance of tiller number in most landraces may indicate lower sensitivity of vegetative growth to water stress. Similar genotype‐dependent responses to drought have also been reported in rice landraces and breeding materials under water‐limited conditions (Mishra et al. 2018; Hussain et al. 2021; Lamichhane et al. 2025).

Two‐way ANOVA showed that shoot length varied significantly among landraces in all agroecological regions, while treatment effects were significant only in the Mid‐hill and Inner‐Tarai regions. In the Tarai region, shoot length was influenced by landrace differences but not by water treatment. Significant reductions were observed in Chamade and Ratomarso under drought conditions; however, there is a lack of significance treatment effect on other landraces based on post hoc analysis. Maintenance of shoot growth under water deficit may support continued biomass production and photosynthetic activity during stress conditions (Zhu et al. 2010). Root length also differed significantly among landraces across all agro‐ecological regions. Treatments were significant only in the Tarai region, and the Anjana landrace showed increased root length under complete drought conditions. Longer root systems may enhance access to deeper soil moisture and contribute to drought adaptation under water‐limited environments (Abd Allah et al. 2010; Kim et al. 2020; Kano et al. 2011). Previous studies have similarly emphasized the importance of root plasticity, biomass allocation, and genotype‐dependent responses in maintaining plant performance under drought stress (Melandri et al. 2021; Panda et al. 2021; Töpfer et al. 2025).

Similarly, root moisture content, shoot moisture content, and shoot‐to‐root ratio showed limited variation across treatments in most landraces, suggesting relative stability of tissue water status and biomass allocation under drought conditions. Maintenance of tissue water balance and biomass partitioning has been associated with physiological stability and improved drought adaptation in rice and other cereals (Pantuwan et al. 2002; Xu et al. 2015).

### 4.2 Reproductive Stability

Our results highlight the resilience of rice landraces in maintaining reproductive performance under drought stress. Panicle length, a key indicator of drought tolerance (Kandel et al. 2022), remained unaffected across all agro‐ecological regions. Similarly, total grains per panicle and the weight of 1000 grains, both crucial components of yield (Nahar et al. 2018; Yadav et al. 2023), were not significantly impacted by drought in most regions, suggesting the ability of these landraces to sustain grain production under water scarcity. However, the Inner‐Tarai region exhibited a reduction in total grains per panicle under drought conditions (Figure 4E), indicating potential vulnerability of some varieties. This reduction in the total grains may be due to comparatively smaller root lengths in the landraces of Inner‐Tarai compared to the shoot length. Despite these variations, the overall stability of panicle characteristics and yield demonstrates the adaptability of these landraces to water stress (Kumar et al. 2023; Panda et al. 2021; Yadav et al. 2023).

The PCA result also shows that there is an insignificant effect of the treatment in their morphological characteristics (Figure 5) but there is landrace‐wise variation; this result could be attributed to the genetic diversity among the varieties. The hierarchical analysis shows the more or less grouping based on the agro‐ecological zones (Figure 6), this highlights the significance of the microclimatic habitats and environmental effect on the landrace characters and their response to the stress conditions or in other words, the place where a landrace comes from affects how it grows. Similar agro‐ecological differentiation and drought‐associated responses among rice landraces have also been observed in studies evaluating mycorrhizal colonization and stress adaptation across water‐limited environments in far‐west Nepal (Dhami et al. 2025). Correlation analysis also showed that the yield is positively correlated with the root length, which is in accordance with the result of Ramamoorthy et al. (2018), but the positive correlation of the yield with the shoot‐root ratio indicates that these rice landraces, which have longer roots but relatively higher shoot biomass, are more resilient to the drought.

The overall resilience of the rice landraces selected in the region is because we choose the rice landraces based on our preliminary survey. Those varieties which are recommended by the farmers are selected for the experiments, and these varieties show excellent resilience, and the perception of the farmers is proved by this experiment scientifically. Farmers already knew that these landraces were good in dry times. Our study used science to test the farmers' perceptions. This is important because it shows that we can learn a lot from farmers' knowledge. This study is significant for two reasons: First, it shows that landraces can be a good choice for farmers to fight against drought. Second, it reminds us that we need to conserve these landraces so we can use their adaptive traits and good qualities in the future.

The stability of reproductive features such as panicle length, grains per panicle, and grain weight in different landraces despite drought stress directly helps to food security. Similarly, strengthening resilience and adaptation capacities to climate‐related threats is emphasized in SDG 13: Climate Action. The ability of these landraces to sustain yield potential during droughts is indicative of their climate change adaptation. Additionally, choosing resilient landraces by combining scientific research and indigenous knowledge highlights a comprehensive strategy to address the effects of climate change. In addition to supporting adaptive farming techniques that are essential for reducing the consequences of climate change, this can improve agricultural sustainability.

## 5 Conclusion

The present study showed that morpho‐reproductive responses to water stress varied primarily among rice landraces, which indicates strong landrace‐specific differences. Although drought reduced tiller number in Anjana and Lalchand, decreased shoot length in Chamade and Ratomarso, and lowered grains per panicle in Jhini and Shanti, other vegetative and reproductive traits including shoot‐to‐root ratio, panicle length, and grain weight remained relatively stable across treatments. PCA and hierarchical clustering further indicated stronger differentiation among landraces than treatments, with partial grouping according to agro‐ecological region. It suggests that there is an influence of local adaptation on trait variation under water‐deficit conditions. Positive associations of yield with root length, grain weight, and tiller number show the importance of biomass allocation and belowground traits in maintaining productivity under limited water availability. Overall, the study identifies the rice landraces with consistent performance across water stress on three agro‐ecological regions of far‐west Nepal. These landraces represent potential genetic resources for future physiological evaluation, conservation, and drought‐focused rice improvement programs under climate‐vulnerable agricultural systems.

## Funding

This work was supported by the University Grants Commission, Nepal (Grant CRG‐77/78‐ S&T‐2).

## Conflicts of Interest

The authors declare no conflicts of interest.

## Data Availability

All the required data is provided in the manuscript. The data generated in this research will be made available upon reasonable request from the corresponding authors.
